# Nephroprotective effect of remote ischemic conditioning on type 2 diabetic rats

**DOI:** 10.22038/ijbms.2024.77896.16855

**Published:** 2024

**Authors:** Seyyed Majid Bagheri, Elham Hakimizadeh, Mohammad Allahtavakoli

**Affiliations:** 1 Physiology-Pharmacology Research Center, Research Institute of Basic Medical Sciences, Rafsanjan University of Medical Sciences, Rafsanjan, Iran; 2 Department of Physiology and Pharmacology, School of Medicine, Rafsanjan University of Medical Sciences, Rafsanjan, Iran

**Keywords:** Diabetes mellitus, Inflammation, Ischemic conditioning, Kidney injury, Oxidative stress

## Abstract

**Objective(s):**

Diabetic nephropathy is one of the main causes of kidney failure in the end stage of diabetes worldwide. The present study was conducted with the aim of using the remote ischemic conditioning (RIC) method to prevent diabetic nephropathy.

**Materials and Methods:**

Diabetes was induced by high-fat diet (60%) and streptozotocin injection (35 mg/kg) in rats. RIC was performed by tightening a tourniquet around the upper thigh and releasing it for three cycles of 5 min of ischemia and 5 min of reperfusion daily for an 8-week duration. At the end of the experiment, serum and urine parameters were examined. Anti-oxidant enzymes and lipid peroxidation levels in the kidney were also determined along with histological examination. The expression levels of tumor necrosis factor-alpha and transforming growth factor beta genes were also evaluated.

**Results:**

Glucose, cholesterol, triglyceride, and HbA1c concentrations were not significantly reduced in the RIC group. On the other hand, serum creatinine, urea, and albumin levels decreased and increased in urine. Anti-oxidant enzymes did improve in the kidney significantly and the expression of tumor necrosis factor-alpha and transforming growth factor beta genes decreased significantly. Histopathological examination also showed that necrosis, epithelial damage, and leukocyte infiltration increased in the diabetic group and improved in the treatment group.

**Conclusion:**

The results of biochemical analysis, and enzymatic and histological examinations showed that although RIC could not reduce blood glucose and lipids, nevertheless it may delay the progression of diabetic nephropathy due to the presence of anti-inflammatory and anti-oxidant activities.

## Introduction

Diabetes is one of the serious metabolic disorders that affects millions of people around the world and its global prevalence in 20–79 year olds in 2021 was estimated to be 10.5% (1). This disease is caused by the lack of insulin secretion or the lack of response of insulin-dependent cells (2). Diabetes mellitus and the resulting hyperglycemia causes the glycosylation of various proteins throughout the body, which causes many complications (3). These complications include damage to the nerves, eyes, liver and is the main cause of chronic kidney failure (4). Diabetic nephropathy (DN) is one of the major threats to diabetic patients, which includes about 40% of end-stage renal diseases (5). The long-term presence of hyperglycemia can lead to abnormal changes in renal hemodynamics and metabolic disorders, which is one of the key factors of kidney damage caused by diabetes (6). Pathologically, DN includes renal structural abnormalities such as increased urinary albumin excretion, increased basement membrane thickening, and cell damage, which are characterized by various mechanisms such as increased oxidative stress, inflammatory responses, and fibrosis (7). To treat this disorder, in addition to lifestyle modification, new treatments are also being developed (8). Studies have shown that the use of some natural compounds (9) or supplements such as zinc and methionine (10) can be effective in reducing kidney damage caused by ischemia-reperfusion. Remote ischemic conditioning (RIC) is a non-invasive procedure in which periods of ischemia and reperfusion are applied to distant organs to protect vital organs by activating endogenous protection (11). This method is derived from *in situ* ischemic conditioning, which was first described by Murray *et al*. The method involves cycles of ischemia and reperfusion that can protect the heart from subsequent sustained ischemic damage (12). The beneficial effects of RICis are caused by various mechanisms such as anti-oxidant activity, reduction of apoptosis, and activation of anti-inflammatory pathways (13). Several studies have investigated the positive effect of RIC in protecting various organs, including the kidney, against ischemia-reperfusion injury (14). Researchers have shown that RIC can exert its protective effects by reducing oxidative stress (15). By investigating the role of RIC in the protection of the kidney caused by ischemia/reperfusion, Oliveira *et al*. showed that RIC is able to reduce the level of MDA and reduce the damage caused by I/R in the kidney (16). Researchers studied the relationship between RIC and anti-oxidant activity in rats by performing three cycles of 5-min ischemia followed by 5-minute reperfusion (17). Their results showed that RIC can increase the anti-oxidant capacity of the liver and kidney. RIC has been shown to protect against I/R injury by reducing key steps leading to systemic inflammation (18). Research has shown that this process blocks NF-κB and subsequently reduces systemic inflammation (19). Among the cytokines linked to inflammatory reactions in type 2 diabetes, transforming growth factor β (TGF-β) has been recognized as a primary factor in DN, which plays an important role in the progression of glomerulosclerosis and interstitial fibrosis (20). Although various studies on the protective effects of this method on the heart and nervous system have been investigated and confirmed (21), so far few studies have been reported on the effects of RIC in protecting kidney tissue against diabetes. In this study, our aim is investigation of the protective effects of RIC method on DN in type 2 diabetic rats.

## Materials and Methods

***Animals***

Twenty four male albino rats with an average weight of 200–250 g were kept in standard conditions with 12 hr of light and 12 hr of darkness and humidity level of 55%. Food and water were provided *ad libitum*. The experiments conducted were carefully approved by the Laboratory Animal Care Committee of Rafsanjan University of Medical Sciences (approval ID of IR.RUMS.AEC.1402.004).

***Remote ischemic conditioning protocol***

RIC was performed by anesthetizing rats with sodium pentobarbital (30 mg/kg) intraperitoneally. RIC was performed by tightening a tourniquet around the upper thigh and releasing it for three cycles of 5 min of ischemia and 5 min of reperfusion daily for a 8 weeks duration (22).

***Experimental procedure***

**Figure 1:**
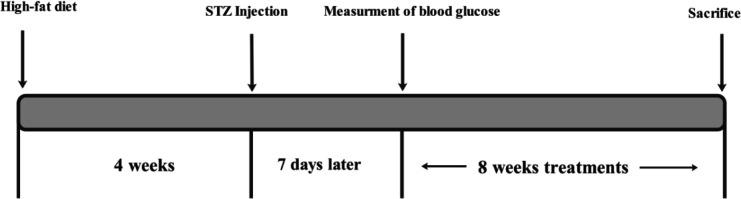
Representative diagram of different experiment steps and their duration

Type 2 diabetes was induced by high-fat diet (60%) for 4 weeks and low dose of STZ (35 mg/kg, IP). Seven days after STZ injection, fasting blood glucose level was measured in all rats, and those with blood glucose above 200 mg/dl were considered diabetic animals (23)^.^ After induction of diabetes, rats were randomly divided into three groups (8 rats in each group): Normal control, diabetic control group, and RIC group (Figure 1). 

***Biochemical analysis of serum***

After taking blood from the orbital sinus of rats, the serum was prepared by centrifugation (3000 rpm, 20 min) and frozen until biochemical analysis. Serum levels of glucose, cholesterol, triglyceride, urea, creatinine, and albumin were measured. HbA1c of plasma also was determined using appropriate kits according to the manufacturer’s instruction. 

***Urine volume and biochemical analysis***

At the end of the experiment, each animal was situated within an individual metabolic cage. Subsequently, urine was collected and its volume was quantified over a span of 8 hr. Samples were utilized to assess the concentrations of urea, creatinine, and micro albumin. 

***Histopathological analysis of kidney***

Kidneys were removed and fixed in 10% neutral formalin. Then all samples were cleaned, dehydrated, and embedded in paraffin. 7-micrometer-thick sections were prepared using a microtome (Leica) and the slides were stained with hematoxylin–eosin (H&E) and histopathological changes were observed microscopically.

***Kidney anti-oxidant parameters***

Biochemical assessments were conducted to determine the levels of oxidative stress biomarkers in the kidney, including glutathione (GSH), superoxide dismutase (SOD), and malonyldialdehyde (MDA). These estimations were carried out using commercially available kits, following the instructions provided by the manufacturer. Additionally, the activity of catalase (CAT) was measured using commercial UV spectroscopic methods. To elaborate, a solution of kidney homogenate supernatant (10 μl) was combined with 0.5 ml of a 10 mM hydrogen peroxide (H_2_O_2_) solution. The resulting mixture was then analyzed for changes in its optical density at a wavelength of 240 nm, using a spectrophotometer. The decrease in optical density observed within a three-minute period after the addition of the kidney homogenate was regarded as an indication of catalase activity.

***Reverse transcription and real-time polymerase chain reaction***

Total RNA was extracted from kidney with RNX-Plus solution (Cinaclone, Tehran, Iran) according to the manufacturer’s instructions. Complementary DNA (cDNA) was synthesized using Thermo Scientific RevertAid first strand cDNA synthesis kit (Prestos, Mashhad, Iran). cDNA through reverse transcription polymerase chain reaction (RT-PCR) was reproduced. Primers used for reverse transcription PCR provided by Betagen Inc. (Mashhad, Iran) were synthesized. β-actin gene was used as an internal control gene, and all the RT-PCR reactions were run in duplicate. The 2^-ΔΔCT^ method was applied to calculate the relative abundance of mRNA transcripts. The sequence of primers is reported in Table 1.

***Statistical analysis***

The results are reported as the mean ± SEM. Differences between means were obtained by one-way ANOVA (Tukey-Kramer method) was performed using Graph Pad prism version 9 software (GraphPad Inc., San Diego, CA, United States).

**Table 1:** Sequence identiﬁcation and primers used for RT-PCR analysis of β-Actin, TGF-β and TNF-α

**No**	**Gen**	**Forward primer **	**Reverse** **primer **
**1**	**β-Actin**	5′‐CGCGAGTACAACCTTCTTGC‐3′	5′GTCTACAACATGATCTGGGTCA3′
**2**	**TGF-β**	5′‐GCAACAATTCCTGGCGTTAC‐3′	5′‐GTATTCCGTCTCCTTGGTTCAG‐3ʹ
**3**	**TNF-α**	5′-GTCGTAGCAAACCACCAAGC‐3′	5′-CTCCTGGTATGAAATGGCAAA‐3′

**Table 2:** Different serum biochemical parameters in normal, treated and diabetic rats

	**Normal**	**Diabetes**	**RIC**
**Glu(mg/dl)**	115±14	366±45^ a^	384±58^ a^
**Chol** **(** **mg/dl** **)**	92±9.2	130±8.8a	123±7.6a
**tg(** **mg/dl** **)**	89±4.5	163±7.2 a	156±41a
**hba1c** **(mg/dl)**	5.6±1.2	18±1.3^ a^	16±1.9^ a^
**Creatinine (mg/dl)**	0.7±0.5	1.8±0.7^ a^	1.1±0.7^ b^
**Urea(mg/dl)**	11.3±1.6	36.5±6.2^ a^	21.5±5.3^ b^
**Albumin (mg/dl)**	3.8±0.5	2.3±0.2^ a^	2.9±0.3^ b^

Data was analyzed by ANOVA followed by Tukey’s test and expressed as the means± SEM. The sample size (n) for each group is 8. a: *P*<0.05 compared to the Normal group and b: *P*<0.05 compared to the diabetic group.

Glu: Glucose; Chol: Cholesterol; TG: Triglyceride

**Table 3:** The effect of Remote ischemic conditioning (RIC) on urinary creatinine, urea and microalbumin levels in diabetic rats

	**Normal**	**Diabetes**	**RIC**
**Creatinine (mg/dl)**	66.3±7.3	23.4±2.4^ a^	40±3.9^ b^
**Urea (mg/dl)**	3.2±0.3	0.8±0.08^ a^	2.1±0.5^ b^
**Micro albumin (mg/dl)**	10.1±1.2	21.1±2.1^ a^	15.1±1.2^ b^

Data was analyzed by ANOVA followed by Tukey’s test and expressed as the means± SEM. The sample size (n) for each group is 8. a: *P*<0.05 compared to the Normal group and b: *P*<0.05 compared to the diabetic group. The sample size (n) for each group is 8.

**Figure 2:**
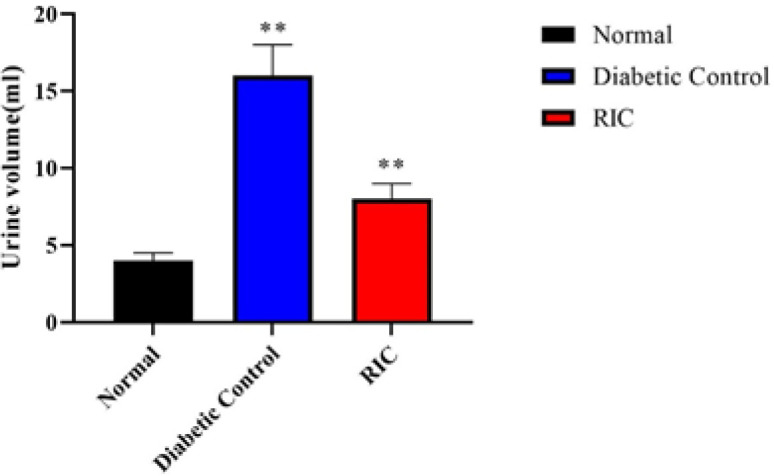
Effects of RIC on urine volume in diabetic rats

Data are expressed as the means ± SEM with a sample size of n=8 per group. ** *P*<0.01 compared to the diabetic group.

RIC: Remote ischemic conditioning

**Figure 3:**
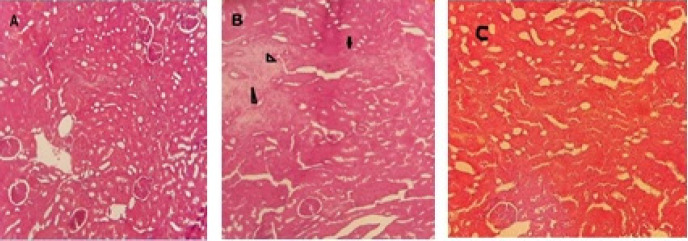
Histopathological changes of kidneys in normal, treated and diabetic rats

The sample size (n) for each group is 8. (a) Control group, (b) diabetic group, disorganization of renal tubule, necrosis (arrow), epithelial damage (arrowhead), and leukocyte infiltration were seen, (c) in RIC group histopathological changes were decreased. H and E, ×100.

RIC: Remote ischemic conditioning

**Figure 4:**
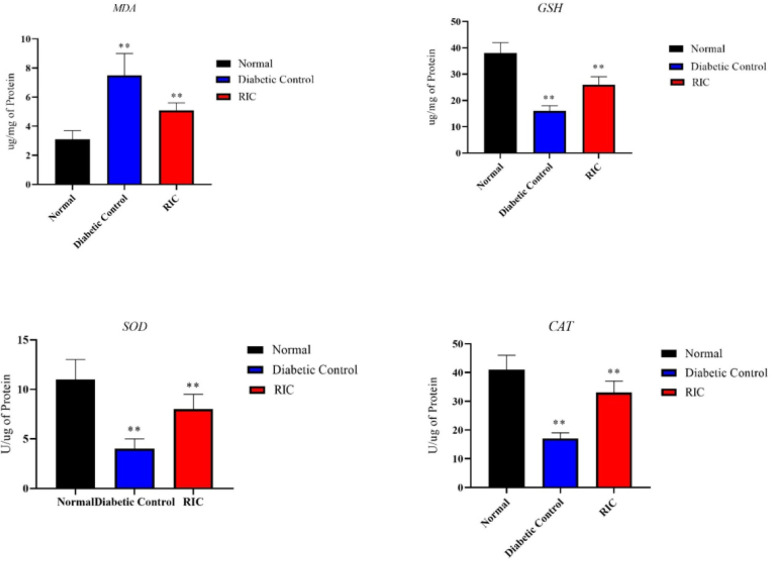
Effects of RIC on superoxide dismutase, catalase, reduced glutathione and lipid peroxidation malondialdehyde in diabetic rats

Data are expressed as the means ± SEM with a sample size of n=8 per group. ** *P*<0.01 compared to the diabetic group

RIC: Remote ischemic conditioning

**Figure 5:**
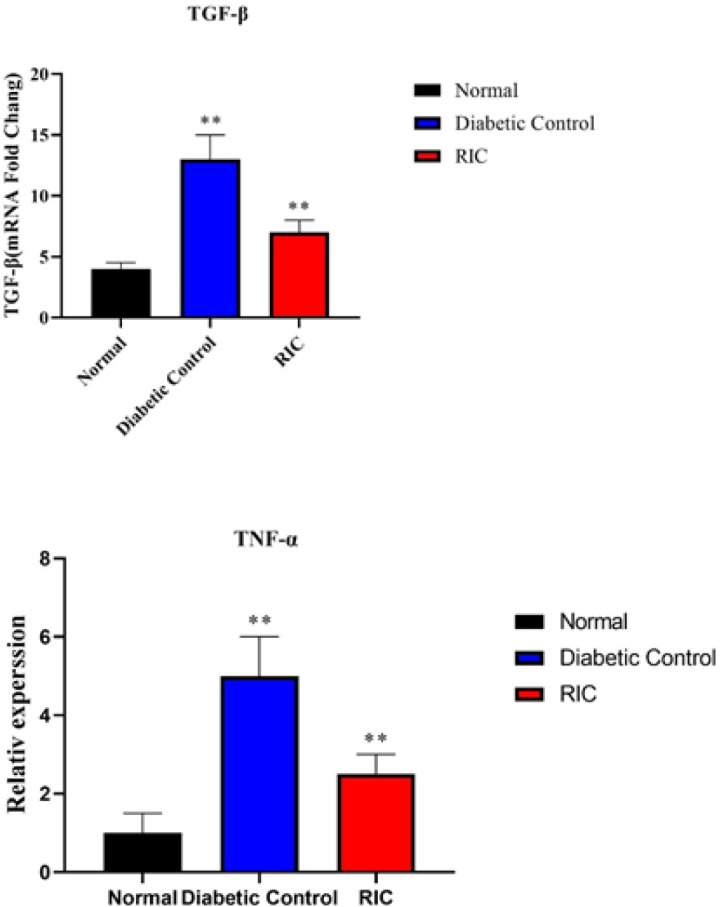
Effect of RIC on the genes expression of TNF-α and TGF-β using RT-PCR in diabetic rats

Data are expressed as the means ± SEM with a sample size of n=8 per group. ** *P*<0.01 compared to the diabetic group.

RIC: Remote ischemic conditioning; TGF-β: Transforming growth factor β

## Results

***Effect of RIC on biochemical of serum***

Within 8 weeks after the induction of diabetes, a significant increase in fasting blood glucose, cholesterol, triglyceride and HbA1c levels was observed in the diabetic group compared to the normal control group. However, diabetic rats treated with RIC did not show a significant decrease in these factors. The level of serum creatinine in diabetic rats increased significantly compared to the normal control group. However, in the treatment group, serum creatinine content decreased significantly. In addition, diabetic rats showed a significant increase in blood urea, while RIC significantly reduced this level. Diabetic rats showed a significant decrease in the serum albumin level, but in the treatment group, the albumin concentration increased significantly compared to the diabetic control group. Tables 2 show the data of the above observations.

***Effect of RIC on volume and biochemical of urine***

At the end of the experiment, measuring the volume of urine produced in different groups showed that the volume of urine in diabetic rats increased significantly within 8 hr compared to the normal group. However, after treatment with RIC for 8 weeks, urine volume was significantly reduced compared with diabetic rats (Figure 2). In addition, diabetic rats had higher levels of creatinine, urea, and micro albumin compared to the normal control group but a significant decrease in creatinine and urea levels was observed in the treatment group compared to the group of diabetic rats (Table 3).

***Renal histopathology***

The kidneys in the control group revealed normal structure with no sign of histopathological changes. The renal glomeruli and tubules was normal (Figure 3a). Histopathology changes in the renal glomeruli and tubules were observed in diabetic group. Disorganization and congestion of renal tubule, necrosis, epithelial damage, and leukocyte infiltration were seen (Figure 3b). In the RIC group, these histopathological changes were decreased but some tubular necrosis was also obvious (Figure 3c). 

***Kidney anti-oxidant parameters***

In the diabetic group, the activities of superoxide dismutase, reduced glutathione and catalase were lower compared to the normal group. However, significantly higher levels of MDA (a marker of lipid peroxidation) were detected in the kidneys in diabetic rats. Treated animals had significantly reduced levels of MDA and increased levels of SOD, GSH, and catalase (Figure 4).

***mRNA expression of TNF-α and TGF-β***

According to the results obtained from the study of the expression level of genes, it was found that in the diabetic group there was a significant increase in the level of TGF-β and TNF-α gene expression compared to the normal group. The obtained PCR results showed a significant decrease in TNF-α as inflammatory marker and TGF-β as the marker of endothelial dysfunction in treated group compared to the diabetic group (Figure 5).

## Discussion

DN is one of the most important complications of diabetes, which is known as the main cause of kidney failure (24). Increased blood glucose level in addition to intensifying oxidative stress and increasing ROS production in mitochondria causes apoptosis in kidney tissue cells (25). Our findings showed that the RIC method has no detectable effect on reducing glucose, triglyceride, cholesterol, and HbA1c. Additionally, it demonstrated that the concentrations of urea, microalbumin, and creatinine in the serum decreased, while these factors increased in urine. Also we observed that the anti-oxidant enzymes and lipid peroxidation in the kidney tissue improved significantly in the RIC group and TGF-β expression decreased compared to the diabetic group. In terms of histopathology, it was also found that the kidney tubule disorganization, necrosis, epithelial damage, and leukocyte infiltration decreased in the RIC group. Both oxidative stress and inflammation play crucial roles in the pathogenesis and advancement of chronic kidney disease (26). Chronic hyperglycemia elicits oxidative stress, facilitates the overproduction of reactive oxygen species (ROS), diminishes the anti-oxidant capacity, and prompts the immune system to secrete inflammatory mediators and cytokines that impact the glomerular capillaries (27). Furthermore, these processes lead to alterations in both the structure and function of the renal tubules, consequently intensifying the damage that occurs in the kidneys and throughout the body (28). RIC refers to a therapeutic approach aimed at protecting organs or tissues against the harmful consequences of ischemic reperfusion injury (29). Although its application was initially demonstrated to provide defense for the cardiac organ against acute myocardial infarction, its advantageous impacts were also observed in other organs such as the brain (30). However, its protective effects against DN remain unexplored. Several investigations have revealed that remote ischemia conditioning acts by activating anti-oxidant, antiapoptotic, and anti-inflammatory pathways (31). It was shown that although RIC does not affect blood glucose levels, it has anti-oxidant properties and can prevent diabetic retinopathy (32). Kong *et al*. demonstrated that ischemic conditioning could reduce expression of IL-1β, TNFα, and ICAM-1, and prevent the accumulation of leukocytes in the cerebral cortex (33). A study showed that RIC temporarily improves anti-oxidant defense and increases both liver and kidney anti-oxidant capacity (17). The ability of ischemia conditioning to increase the anti-oxidant capacity of the brain has been confirmed (34). TGF-β causes extracellular matrix thickening, hypertrophy, and increased collagen production in mesenchymal cells (35). Transforming growth factor β is involved in the development of glomerulosclerosis and interstitial fibrosis in DN (36). Studies have shown that RIC exerts its protective effects through humoral mediators, neural mechanisms, or their combination. It is known that RIC can cause the release of mediators such as kallistatin, apolipoprotein A-I, and stroma-derived factor 1α (SDF-1α)(37). Kallistatin is a protease that reduces inflammation, apoptosis, and oxidative stress in endothelial cells(38). Apolipoprotein A-I also has anti-inflammatory properties and can prevent ischemia-reperfusion injury (39). Several other potential mediators such as micro RNAs (miRNAs), bradykinin, adenosine, and nitric oxide (NO) can prevent ischemia-induced tissue damage (40). On the other hand, in addition to the humoral hypothesis, there is also the neural reflex hypothesis, which states that an ischemia-reperfusion cycle in peripheral locations may activate a neural reflex and lead to organ protection (40). Activation of afferents during RIC occurs due to local accumulation of mediators such as calcitonin gene-related peptide (CGRP), adenosine, and bradykinin. These mediators cause neural metaboreflexes, and RIC is a conditioning protocol that induces these neural metaboreflex (41). However, more evidence and studies are needed to know the effective mechanisms in this process so that this method can be used to reduce the diabetic complications.

## Conclusion

RIC is a relatively new method that has shown its protective effects on various tissues. However, understanding the mechanisms of this method in the treatment of diseases is still challenging. The results showed that RIC can reduce DN in diabetic rats by improving anti-oxidant capacity and reducing inflammatory pathways. Although this method could not reduce blood sugar, it showed that it is able to protect the kidney against the damage caused by diabetes. Future studies should focus on the use of this method in the treatment of diabetic patients so that its effectiveness in the treatment of diabetes can be used. There were some limitations in this research. Due to low financial resources, it was not possible to investigate other remote ischemia conditioning mechanisms such as apoptotic factors.
